# Transforming Growth Factor-β/Smad Signaling Inhibits Melanoma Cancer Stem Cell Self-Renewal, Tumor Formation and Metastasis

**DOI:** 10.3390/cancers16010224

**Published:** 2024-01-03

**Authors:** Julien Boudreault, Ni Wang, Mostafa Ghozlan, Jean-Jacques Lebrun

**Affiliations:** Cancer Research Program, Department of Medicine, Research Institute of McGill University Health Center, Montreal, QU H4A 3J1, Canada; julien.boudreault2@mail.mcgill.ca (J.B.); ni.wang79@gmail.com (N.W.); mostafa.ghozlan@mcgill.ca (M.G.)

**Keywords:** TGFβ, melanoma stem cells, tumor formation, metastasis

## Abstract

**Simple Summary:**

Transforming growth factor-beta (TGFβ) mediates various biological processes including cell growth, cell death, cellular differentiation and stemness, among others. TGFβ also regulates tumor formation and metastasis in a context-dependent manner. This research aims to investigate and define the role of the TGFβ cell signaling pathway in melanoma, which is a deadly form of skin cancer. Using relevant melanoma cancer cell lines and preclinical models of melanoma, we show that TGFβ acts as a potent tumor suppressor and negative regulator of cancer stemness and metastasis in melanoma. These findings will be instrumental for the future development of targeted therapy in melanoma.

**Abstract:**

The secreted protein transforming growth factor-beta (TGFβ) plays essential roles, ranging from cell growth regulation and cell differentiation in both normal and cancer cells. In melanoma, TGFβ acts as a potent tumor suppressor in melanoma by blocking cell cycle progression and inducing apoptosis. In the present study, we found TGFβ to regulate cancer stemness in melanoma through the Smad signaling pathway. We discovered that TGFβ/Smad signaling inhibits melanosphere formation in multiple melanoma cell lines and reduces expression of the CD133+ cancer stem cell subpopulation in a Smad3-dependent manner. Using preclinical models of melanoma, we further showed that preventing Smad3/4 signaling, by means of CRISPR knockouts, promoted both tumorigenesis and lung metastasis in vivo. Collectively, our results define new functions for the TGFβ/Smad signaling axis in melanoma stem-cell maintenance and open avenues for new therapeutic approaches to this disease.

## 1. Introduction

Melanoma is a malignant tumor of melanocytes which typically arises from the skin. Despite recent progress in targeted therapies, melanoma has the highest death tolls among all skin cancer types [1]. Patients diagnosed with early stage melanoma (I–III) can have their skin tumors removed surgically with high success [2]. However, high plasticity and metastatic capacity in later stages (IV) of aggressive melanoma is linked with poor prognosis [3]. A major challenge in the treatment of melanoma originates from the multiple levels of heterogeneity of this disease [4]. 

Multiple mutations in the BRAF, NRAS, NF1, PTEN, KIT, TP53 and hTERT genes have been reported in melanoma [5]. Several other signaling pathways are also often mutated in cutaneous melanoma, including PI3K/AKT [6], Wnt [7], NF-κB [8], Jnk [9], JAK/STAT [10] and TGFβ [11]. In particular, previous work from our laboratory and others revealed that TGFβ acts as a strong tumor suppressor and inhibits cell growth, migration and invasion in melanoma [12,13,14]. The TGFβ signaling pathway is activated through ligand binding on its membrane receptors, triggering their serine–threonine protein kinase activity. After the subsequent recruitment and phosphorylation of TGFβ central downstream effectors, Smads then initiate the signal transduction cascade. Smads act as transcription factors and regulate the expression of the multiple TGFβ target genes regulating its tumor-suppressive effects, including inhibition of cell proliferation, induction of apoptosis and suppression of cell immortalization [15,16]. 

Tumors possess a hierarchical organization of cells and contain stem-like cells, which are responsible for sustaining tumor growth [17,18]. These cancer stem cells (CSCs) represent a rare subpopulation of the bulk of the tumor that possesses self-renewal capacities and exhibits high resistance to conventional treatments. Such plastic and resilient cells have propagating functions that are essential for primary tumor growth and metastasis dissemination [19]. The embryonic origin of melanocytes, from which melanoma arises, comes from the neural crest stem cell [20]. Comparable to other types of CSCs, melanoma CSCs can initiate new tumors and regenerate the heterogeneous cancer cell populations of the bulk of the tumor [21]. Several cell-surface markers have been linked to melanoma CSCs’ self-renewal capacity, including ABCG2 [22], ABCB5 [23], ALDH [24,25], CD133 [22], CD20 [21], CD166 [26], CD271 [27] and Nestin [26]. CSCs reside and interact with the surrounding microenvironment, called the ‘niche’, via secreted factors and molecular signals maintaining their sustainability and maintenance [28]. One of such factors, TGFβ, has been linked with the regulation of cancer stem-cell maintenance in different types of cancers [19,20,21,22,23,24,25,26,27,28,29,30]. However, a role for TGFβ in regulating stemness in melanoma has yet to be uncovered and established.

Considering the strong anti-tumorigenic effects of TGFβ in melanoma, we hypothesized that the TGFβ signaling pathway could play a role in regulating melanoma stemness as part of its tumor-suppressive activities. In this study, we found that TGFβ inhibits melanoma stem cell maintenance in various cutaneous melanoma cell lines originated from different patients. We showed that TGFβ can inhibit melanoma tumorsphere formation and reduce the CD133+ melanocytic stem cell population. We further show that these effects are mediated through the Smad pathway and that Smad3/4 gene silencing by means of CRISPR/Cas9 knockout (KO) could prevent the TGFβ anti-stemness effects in melanoma. Moreover, using preclinical models of melanoma, we showed that the orthotopic transplantation of Smad3/4 CRISPR-KO melanoma cells led to a significant increase in tumor growth and lung metastatic nodule formation in vivo, further highlighting the strong tumor-suppressive role of TGFβ in melanoma. Together, these results define a new role for the TGFβ/Smad signaling axis in stem-cell maintenance in melanoma and open avenues for the development of new therapeutic approaches to this deadly disease. Indeed, clinical approaches aiming at stimulating TGFβ signaling could prove useful to improve melanoma patient outcome, including patients with both primary and secondary metastatic tumors. 

## 2. Materials and Methods

### 2.1. Reagents and Chemicals

Recombinant human transformation growth factor beta (TGFβ), epidermal growth factor (EGF), and fibroblast growth factor-basic (b-FGF) were purchased from Peprotech (Ville-St-Laurent, QC, Canada); puromycin, tissue culture medium RPMI1640, DMEM, fetal bovine serum, and B-27™ Plus Supplement (50X) Catalog were purchased from GIBCO (Waltham, MA, USA).

### 2.2. Cell Lines

Cutaneous melanoma cell line WM793B was isolated from the primary tumors of a 37-year-old male patient and is mutant for BRAF (V600E and W274X), PTEN (homozygous deletion) and CDK4 (K22Q). WM278 cell lines were isolated from a 62-year-old female patient and are mutant for BRAF (V600E) and PTEN (hemizygous deletion). A375m, the metastatic variant of A375, was isolated from a 54-year-old patient having an amelanotic melanoma cancer and is BRAF (V600E) and CDKN2A (E61X and E69X) mutant. The BLM cell line, mutant for NRAS (Q61R), was obtained from the lung metastasis of the BRO melanoma cell line, which comes from a 34-year-old male. WM793B, WM278, BLM and A375m were kindly provided by Dr Alan Spatz and Mounib Elchebly (McGill University, Montreal, QC, Canada). DAUV (also called LB33-MEL.A) was derived from a subcutaneous metastatic lesion (stage IV) in a 42-year-old female patient (WT for BRAF and NRAS). The DAUV cell line was generously provided by Dr. Louise Larose (McGill University, Montreal, QC, Canada). RPMI medium supplemented with 10% FBS is used for the 1205Lu, DAUV, MALME-3M, WM278 and WM793 cell lines. DMEM medium supplemented with 10% FBS was used for the A375m and BLM cell lines.

### 2.3. CRISPR Knock-Out

LentiCRISPRv2 (Addgene, cat. No. 52961) was digested using the Esp3I restriction enzyme (ThermoFisher, cat. No. ER0451, Toronto, ON, Canada), dephosphorylated using FastAP (ThermoFisher, cat. No. EF0654), agarose gel purified and extracted using a QIAquick Gel Extraction Kit (QIAGEN, cat. No. 28704, Germantown, MD, USA). Each single-guide primer sequences shown in Table 1 (5′-3′) was phosphorylated using T4 PNK (NEB, cat. No. M0201S, Beverly, MA, USA), annealed by slow cooling from 65 °C to room temperature in T4 ligation buffer (NEB, cat. No. B0202S) and ligated in Esp3I-digested lentiCRISPRv2 purified plasmid using Quick Ligase (NEB, cat. No. M2200S). Each sgRNA ligated plasmid was transformed in STBL3 chemically competent *E. coli* (ThermoFisher, cat. No. A10469) and collected from an amplified single bacterial colony using a QIAprep Spin Miniprep Kit (QIAGEN, cat. No. 27104) [31,32].

Each sgRNA was designed with ChopChop [33]. The chromosomal positioning of the sgRNA binding site as well as off-target and on-target activity evaluation was performed with CRISPOR [34] (Supplementary Table S1). 

### 2.4. qPCR

Total RNAs were extracted using Trizol ^TM^ (Invitrogen, Carlsbad, CA, USA; ThermoFisher Scientific, Waltham, MA, USA) as per the manufacturer’s instructions. Then, 2 μg of RNA was reverse transcribed using M-MLV reverse transcriptase and random primers (Invitrogen, Carlsbad, CA, USA) as per the manufacturer’s protocol. The amplification of cDNA was performed by quantitative real-time PCR (qPCR) SsoFast™ EvaGreen^®^ Supermix (Bio-Rad, Hercules, CA, USA) using a Rotor-Gene™ 6000 Real-time Analyzer (Corbett Life Sciences, Germantown, MD, USA), and data were analyzed with its corresponding software. The qPCR conditions were: 30 s at 95 °C, then 40 cycles of 5 s at 95 °C, 5 s at 60 °C and finally 5 s at 72 °C. Human GAPDH was used as a housekeeping gene. Primer sequences are listed in the Table 2.

### 2.5. Lentivirus Production and Cell Infection

HEK293T cells were cultured to 90% confluence in complete medium and transfected with respective lentiCRISPRv2 scramble (scr), Smad2, Smad3 or Smad4 constructs or shRNAS non-targeting control (NTC) and SMAD3 (Sigma-Aldrich, St. Louis, MO, USA) lentiviral packaging plasmids pMD2.G (Addgene#12259) and ps.PAX2 (Addgene #35002), Opti-MEM medium (Invitrogen) and bPEI (Sigma-Aldrich). Medium enriched in virus particles was collected after 48 h. Cells were grown to 50% confluence in antibiotic-free medium in 6-well plates; each well was infected with 100 μL of lentiviruses in the presence of polybrene (hexadimethrine bromide) at 8 μg/mL. For BLM and WM278, cells were infected by spinfection (2 h, 1500 G and 33 °C), and the medium was replenished immediately after centrifugation. For the a375m cell line, incubation was made overnight and replenished with fresh complete medium for 48 h. Cells were selected with 1 μg/mL puromycin 2 days post-infection. The pool of resistant cells forming the stable CRISPR knockout cells was expanded in complete medium supplemented with 1 μg/mL puromycin.

### 2.6. Western Blot Assays

Cells were lysed in ice-cold full lysate buffer (10 mM Tris-HCl, pH 7.5, 5 mM EDTA, 150 mM NaCl, 30 mM sodium pyrophosphate, 50 mM sodium fluoride, 1 mM sodium orthovanadate, 1% Triton X-100, 1 mM phenylmethylsulfonyl fluoride, 10 μg/mL leupeptin hydrochloride, 10 μg/mL aprotinin, 10 μg/mL pepstatin and 10X Phosstop (Sigma-Aldrich). Total protein lysates were quantified using a Pierce™ BCA Protein Assay Kit (Thermo Scientific). Lysates containing 50 μg of total protein were separated by SDS-PAGE, transferred to a nitrocellulose membrane using a wet transfer tank system and probed using specific primary antibodies and HRP-conjugated secondary antibodies. The primary antibodies used for Western blot analysis were a rabbit polyclonal Smad2/3 antibody (Santa Cruz Biotechnology, D767, Santa Cruz, CA, USA), Smad4 antibody (EMD millipore, MAB1132, Billerica, MA, USA), and B-Tubulin (Cell signaling, 2146S, Danvers, MA, USA).

### 2.7. Flow Cytometry

Monolayer cells were dissociated, washed once in ice-cold PBS, resuspended in FACS buffer (PBS, 2% FBS) and counted using a TC20™ Automated Cell Counter (Bio-Rad). Cells were aliquoted at a density of 0.25 × 10^6^–1 × 10^6^ cells per tube. R-phycoerythrin (PE) Mouse Anti-Human CD133 antibody (Miltenyi Biotec) was added to the cell suspension in a ratio of 1:20 (*v*/*v*), gently mixed with cells by gentle flicking and incubated on ice protected from light using an aluminum foil tube covering for 30 min. Samples were washed twice with FACS buffer and analyzed with a BD FACSCanto flow cytometer (BD Biosciences, San Jose, CA, USA)) using excitation of 488 nm and emission using a 575/26 bandpass filter (BD Biosciences). Data were analyzed with FlowJo software (Tree Star Inc., Ashland, OR, USA).

The CD133+ population was analyzed using an anti-CD133 antibody (Miltenyi Biotec™, Bergisch Gladbach, Germany) and the ALDH+ population was analyzed by assessing the enzymatic activity of ALDH with non-immunological ALDEFLUOR™ kit (STEMCELL technologies, Vancouver, BC, Canada). Unstained cells were used to gate the population of CD133+, while ALDH was gated based with an enzymatic ALDH inhibitor, N,N-diethyl-amino-benzaldehyde (DEAB), which was used to block all ALDH isoenzymes activity.

### 2.8. Melanosphere Culture Assay

Melanoma cells were seeded at a density from 5000 to 10,000 cells per well in ultra-low-attachment 24-well plates (Corning, Corning, NY, USA) in 1 ml of freshly prepared stem cell medium (serum-free RPMI1640 or DMEM medium supplemented with 10  ng/mL EGF, 10 ng/mL bFGF and 1X B-27™ Plus Supplement). Low-attachment plates were incubated continually without handling and disruption for 7 days at 37 °C with 5% CO_2_. Spheroids from both passages of a diameter ≥ 50μm were counted as melanospheres.

### 2.9. In Vivo Studies

Mice housing and handling was made in accordance with the approved guidelines of the Canadian Council on Animal Care and the Animal Care Committee of McGill University (AUP # 7497). The immune-deficient non-obese diabetic scid gamma (NSG) mouse breeders were purchased from The Jackson Laboratory.

The human melanoma cancer cell line a375m (1 × 10^6^/mouse) was inoculated in 7-week-old male NSG mice by subcutaneous injection to generate melanoma tumors. The mice were euthanized at the indicated endpoint time, and the tumor size was measured with a digital electronic caliper three times per week. To generate a growth curve, tumor volumes were calculated according to the following formula:

$$\frac{4}{3}\times \pi \times \left(\frac{Length}{2}\right)\times {\left(\frac{Width}{2}\right)}^{2}$$

Human melanoma cancer cell line a375 m (5 × 10^5^/mouse) was injected by tail vein to allow for lung metastasis development. The mice were euthanized 15 days post-injection. The lung tissues were fixed with Bouin’s solution, and metastatic nodules were counted using a microscope.

## 3. Results

### 3.1. TGFβ Inhibits Stem-Cell Maintenance and CSC Self-Renewal Capacity in Melanoma

**Figure 1:**
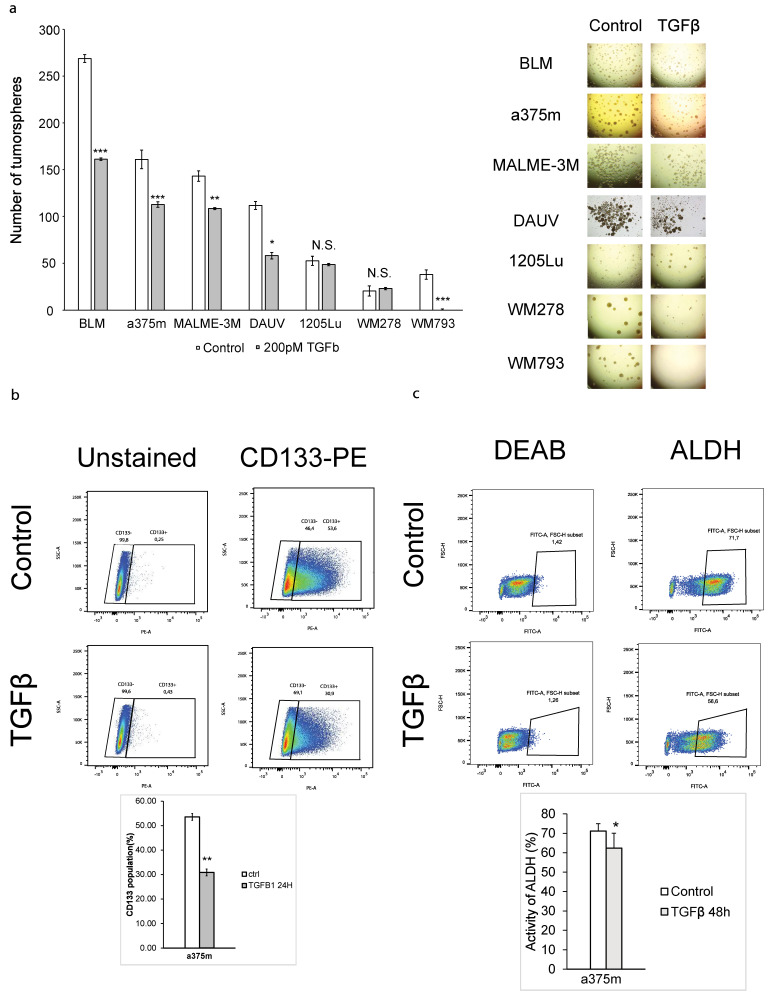
TGFβ inhibits tumorsphere formation and self-renewal capacity in melanoma. (**a**) TGFβ effects on tumorsphere formation of different melanoma cell lines. Left panel: Histogram showing the number of tumorspheres. Right panel: Representative images of tumorspheres of each melanoma cell line. (**b**) Histogram of flow cytometry analysis of a375m cells untreated or treated with TGFβ (200 pM) for 24 h and labeled with a PE-conjugated anti-CD133 antibody. The percentage of CD133-positive/negative populations of a replicate is represented in the dot plot. Gating was set by unstained samples. (**c**) Histogram of flow cytometry analysis of a375m cells untreated or treated with TGFβ (200 pM) for 48 h and evaluated with enzymatic assay ALDEFLUOR™ kit (STEMCELL technologies, Vancouver, BC, Canada). Data are expressed as mean ± standard error. * *p* ≤ 0.05, ** *p* ≤ 0.01, *** *p* ≤ 0.001, n.s. not significant.

The role of TGFβ on CSC stemness remains to be fully investigated and appears context dependent, as TGFβ can either inhibit or sustain CSC maintenance [19]. TGFβ has been reported to regulate CSCs in breast cancer [30,35,36,37], glioblastoma [38,39], gastric carcinoma cells [40], and squamous carcinoma stem cells [40]. Despite its potent tumor-suppressive role in melanoma, the effect of TGFβ cancer stemness has not been addressed yet in these tumors. To first address this, we examined the TGFβ effects in vitro using a melanoma tumorsphere-forming assay (TFA) [21]. TFAs are standard assays used for tumor-initiating capacity measurement and self-renewal assessment [30,37]. We investigated a panel of 7 different human cutaneous melanoma cell lines with various clinical backgrounds (WM278, WM793, a375m, BLM, MALME-3M, 1205Lu and DAUV). We found that TGFβ1 significantly reduced melanoma tumorsphere formation at picomolar concentrations in all cell lines tested except WM278 and 1205Lu (Figure 1a). These effects were particularly striking in WM793 cells where TGFβ stimulation led to a complete inhibition of tumorsphere formation. While the WM278 cell line showed no statistical difference in the reduction in the number of tumorspheres, they exhibited a smaller tumorsphere size (Figure 1a). This consistent suppression of non-adhesive sphere formation across various cell lines suggests a mechanism where TGFβ inhibits CSC self-renewal capacity in melanoma. 

To further investigate the function of TGFβ on melanoma cancer stemness, we measured its effects on two well-characterized melanoma CSC markers: expression of the cell-surface marker CD133 [22] and aldehyde dehydrogenase (ALDH) enzymatic activity [24,25]. Indeed, cells with high CD133 (CD133+) expression [22] or high ALDH enzymatic activity (ALDH+) exhibit increased tumor burden when transplanted in immunodeficient mice, which is in correlation with high CSC self-renewal properties [22]. In silico TCGA analysis further revealed that melanoma tumors are enriched in ALDH1A1 and ALDH1A3 isoenzymes [41]. We thus investigated whether TGFβ could modulate CD133+ and ALDH+ populations in A375m melanoma cells, using flow cytometry. A375m melanoma cells are enriched in the CD133+ population and exhibit a high metastatic potential, and as such, this cell line represents an ideal model to study melanoma stemness. As shown in Figure 1b,c, TGFβ decreased the percentage of both CD133+ and ALDH+ CSC subpopulations.

**Figure 2:**
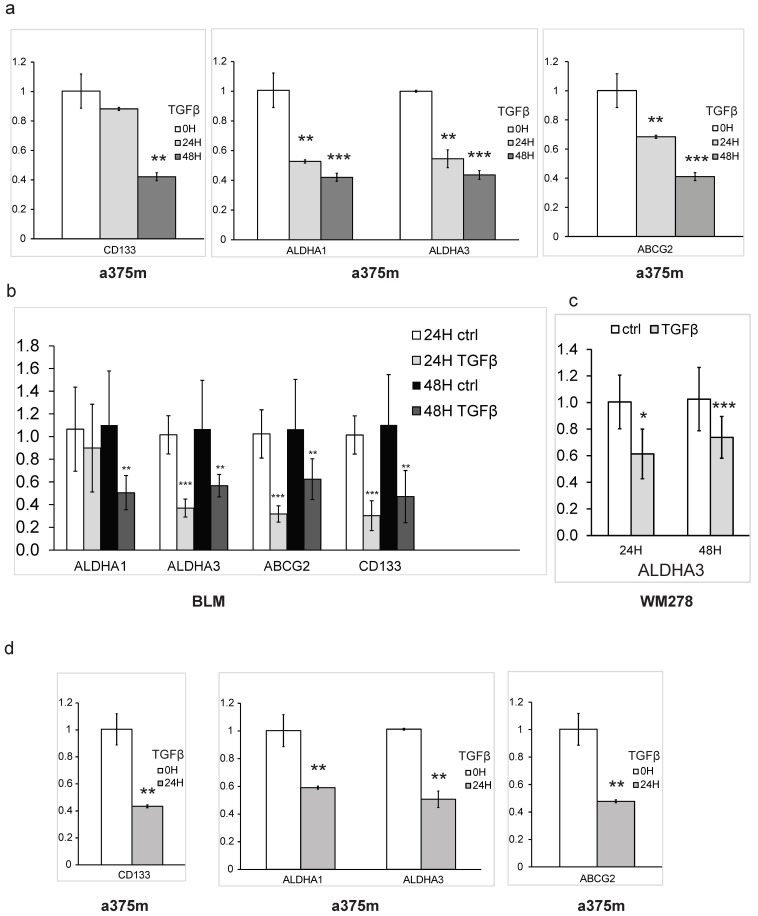
Transcriptional downregulation of stemness markers by TGFβ in melanoma: (**a**–**c**) histogram of relative mRNA expression measured by qPCR of cells collected from (**a**) a375m, (**b**) BLM and (**c**) WM278 cultured in monolayer condition and a375m in tumorsphere condition (**d**). Cells were exposed to TGFβ (200 pM) for 24 h or 48 h. Data represent ± SEM of triplicate experiments. Data are expressed as mean ± standard error. * *p* ≤ 0.05, ** *p* ≤ 0.01, *** *p* ≤ 0.001.

To obtain further insights into the mechanism by which TGFβ regulates markers implicated in melanoma stemness, we examined the TGFβ-mediated regulation of specific melanoma CSC markers (ALDHA1, ALDHA3, CD133 and ABCG2) at the transcriptional level. As shown in Figure 2a,b, the exposure of a375m and BLM cells to picomolar concentrations of TGFβ significantly reduced mRNA expression of all four CSC markers in a time-dependent manner. TGFβ-mediated decreases in ALDHA3 expression were also observed in a third melanoma cell line (WM278), as shown in Figure 2c. We then assessed the TGFβ effects on the expression of these CSC markers using a more relevant 3D culture model, which better represents the morphology and heterogenous aspects of the tumor biology [42]. Interestingly, TGFβ stimulation of the cells also led to a significant and strong decrease in the CSC markers in tumorsphere conditions (Figure 2d). Altogether, these results define a new function for TGFβ in regulating stem cell maintenance in melanoma and highlight its strong inhibitory effects on CSC self-renewal activity and cell surface CSC marker expression.

### 3.2. The Smad3/4 Pathway Is Required for TGFβ-Mediated Inhibition of Melanoma Cancer Self-Renewal

**Figure 3:**
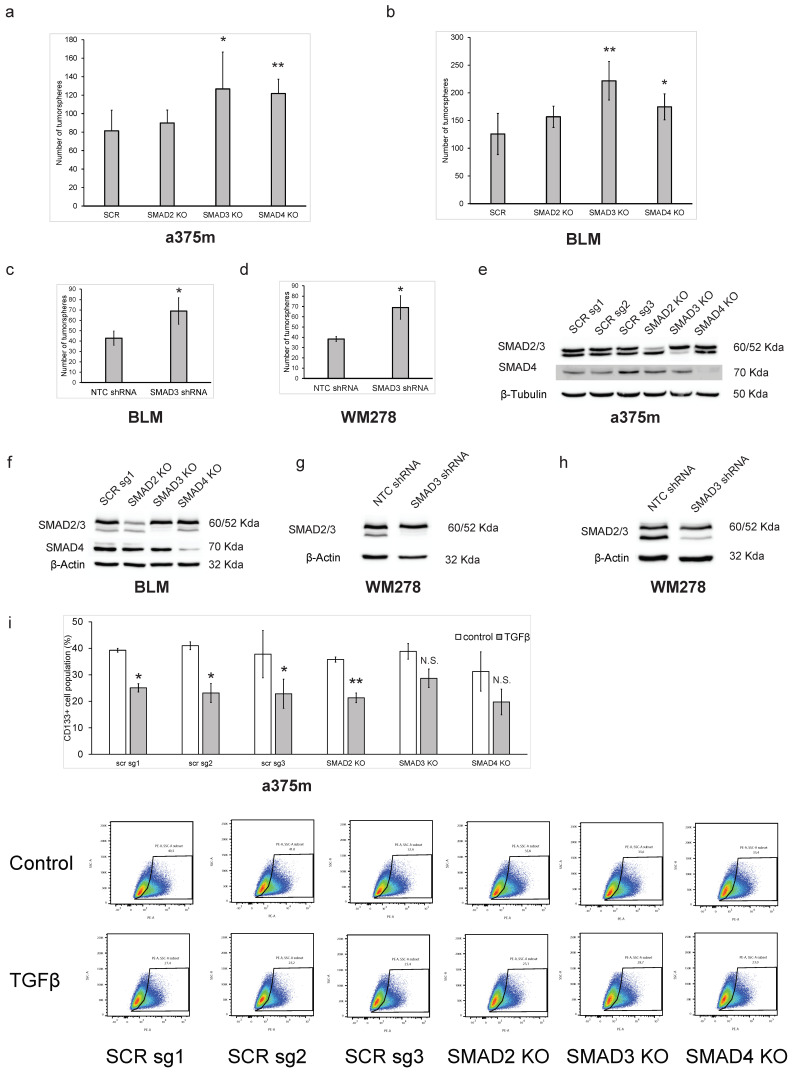
The Smad3/4 pathway is required for TGFβ-mediated inhibition of melanoma cancer stemness. Histograms showing the number of tumorspheres after 7 days culture under low-attachment conditions with CRISPR-Smad2, 3, 4 KOs in (**a**) a375m and (**b**) BLM cell lines or Smad3 shRNA knockdown in (**c**) BLM and (**d**) WM278 melanoma cell lines. High efficiency of the (**e**,**f**) CRISPR KOs and (**g**,**h**) shRNA knockdown was ensured by Western blot. (**i**) Histogram of flow cytometry analysis of different CRISPR KOs produced in a375m cells untreated or treated with TGFβ (200 pM) for 24 h and labeled with a PE-conjugated anti-CD133 antibody. Gating was set by unstained samples. The percentage of CD133-positive/negative populations is indicated. Data are expressed as mean ± standard error. * *p* ≤ 0.05, ** *p* ≤ 0.01 and n.s. not significant. The uncropped blots are shown in Supplementary Materials.

The main signaling pathway activated downstream of TGFβ is the canonical Smad pathway. In particular, Smad2, 3 and 4 play a central role in mediating the TGFβ tumor-suppressive activities in multiple types of cancer [16]. To address whether the canonical Smad pathway is involved in the mediation of the TGFβ effects on melanoma self-renewal, we generated specific Smad2, Smad3 and Smad4 knockout (KO) in two different melanoma cell lines, A375m and BLM, using CRISPR genomic editing. Specific guide RNAs (gRNAs) were designed for each Smad, as described in the methods. Non-targeting scrambled (scr) gRNAs were used as negative controls. Interestingly, we found that blocking Smad3 and Smad4 gene expression but not Smad2 significantly increased melanoma tumorsphere formation in both cell lines (Figure 3a,b). The efficiency of the Smad CRISPR KOs was verified by Western blot and showed near complete inhibition of their respective targets (Figure 3e,f). To further broaden the scope of our findings and further strengthen our results, we also used a parallel shRNA approach to knockdown Smad3 gene expression in BLM cells as well as in a third melanoma cell line (WM278). A non-targeting (NT) gRNA was used as the negative control. As shown in Figure 3c,d, blocking Smad3 expression also resulted in a significant increase in tumorsphere formation in both cell lines, which is consistent with the data obtained with the CRISPR Kos. The high efficiency of the Smad3 shRNA knockdown was verified by Western blot (Figure 3g,h). The increased tumorsphere numbers observed when blocking Smad3 and Smad4 expression likely reflects the inhibition of basal Smad signaling resulting from autocrine TGFβ activity in these cells. Indeed, in melanoma cells, constitutive SMAD signaling occurs in response to autocrine TGFβ secretion [43].

We further analyzed the TGFβ effects on CD133 expression in the different Smad-KOs, using flow cytometry. As shown in Figure 3i, we also found that blocking Smad3 and Smad4 significantly reversed the TGFβ inhibitory effect on CD133 expression. Smad2 gene silencing showed no significative effect on the TGFβ response, which was consistent with the result obtained in the tumorsphere assay. These results indicate that TGFβ-mediated regulation of CSC self-renewal capacity and possible stemness maintenance is Smad-dependent but also specific to the Smad3/4 pathway. 

### 3.3. Blocking TGFβ/Smad Signaling Promotes Melanoma Tumor Growth In Vivo

**Figure 4:**
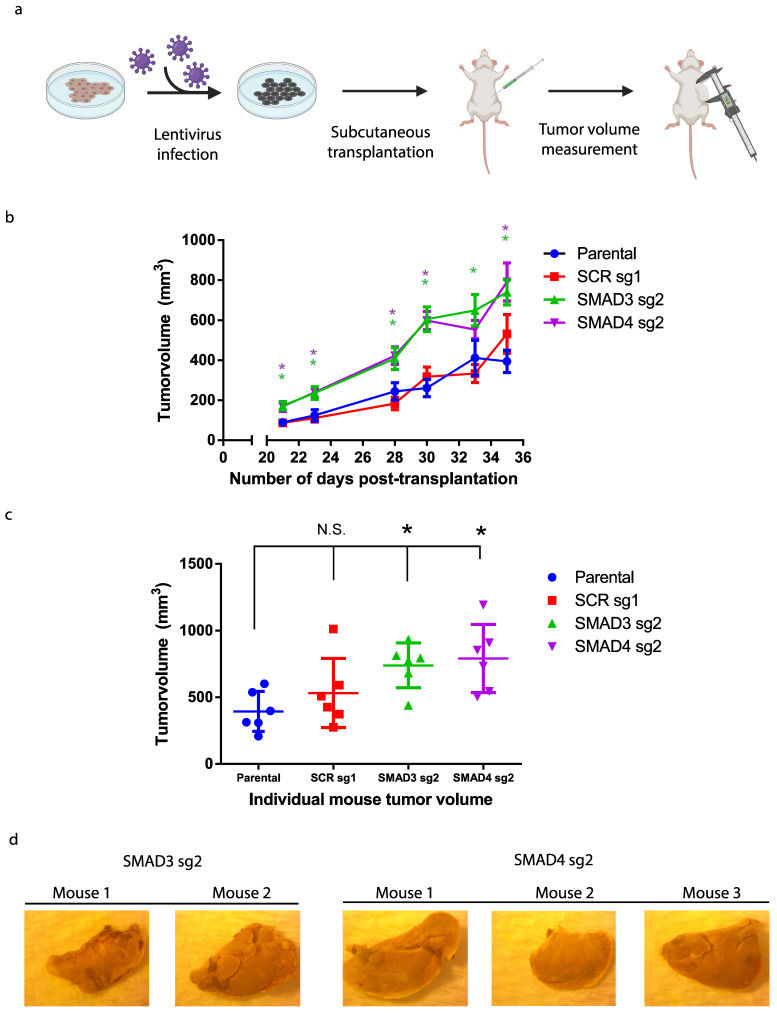
Blocking TGFβ/Smad signaling promotes melanoma tumor growth in vivo. (**a**) Graphical abstract of the orthotopic subcutaneous transplantation of melanoma cells in NSG mice (*n* = 6 per group). (**b**,**c**) One million CRISPR KO a375m cells were transplanted in NSG mice. Tumor growth was assessed by measuring tumor volume 3 times/week (**b**) and at endpoint (**c**). Data are represented as mean ± SEM. *p* values are comparing each KO group vs. scramble control by a two-sided unpaired *t* test at the same day. * *p* < 0.05, n.s. not significant. SMAD3 sg2 is in green and SMAD4 sg2 is in purple. (**d**) Representative pictures of spontaneous metastasis in resected liver by Blouin staining.

Having shown that Smad3/4 gene silencing promote stemness and increases tumorsphere formation, and considering the prominent role played by cancer stem cells in promoting tumor formation, we next assessed the Smad3/4 CRISPR Kos in vivo, using preclinical models of melanoma tumor formation. Orthotopic subcutaneous human tumor xenografts were performed in NOD-SCID IL2Rγnull (NSG) mice. A total of 4 groups of NSG mice (7 mice/group) received a subcutaneous injection of the non-targeting control, Smad3 and Smad4 CRISPR KOs, generated in the A375m melanoma cell line (Figure 4a). Interestingly, blocking the Smad signaling pathway, by means of Smad3/4 CRISPR KO, significantly increased both tumor volume (Figure 4b) and tumor mass (Figure 4c) compared to the non-targeting control (scrambled) and parental cell (A375m) groups. The observed increase in primary melanoma tumor growth upon the depletion of Smad proteins demonstrates their crucial role in suppressing tumorigenicity in vivo, further highlighting the strong tumor-suppressive role played by the TGFβ signaling pathway in melanoma. 

Moreover, while no mice from the parental and scrambled KO groups harbored any secondary metastatic tumors, several mice in both the Smad3 and Smad4 groups developed spontaneous liver metastasis (Figure 4d). These results suggest that the TGFβ/Smad signaling axis not only acts as a potent tumor suppressor but also as a suppressor of metastasis.

### 3.4. The TGFβ/Smad Pathway Inhibits Melanoma Lung Metastasis In Vivo

**Figure 5:**
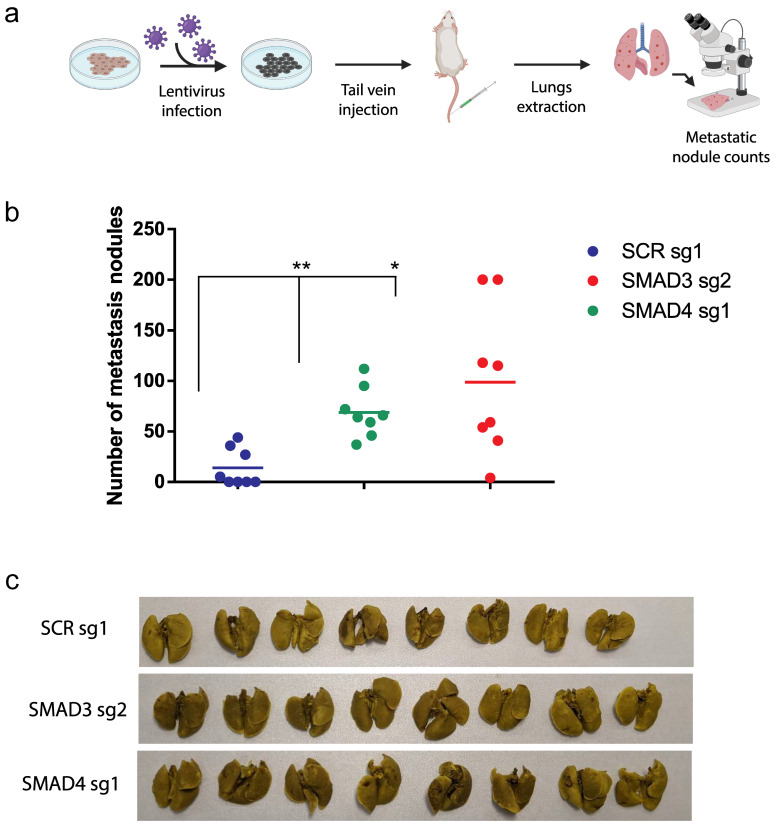
The TGFβ/Smad pathway inhibits melanoma lung metastasis in vivo (**a**) SCR, SMAD3 and SMAD4 KO a375m melanoma cells were injected intravenously in the tail vein of NSG mice (*n* = 8 per group) to assess the number metastatic nodules in the lungs. Data are represented as dot plots for individual mice. The midlines show median value. Data are expressed as mean ± standard error. * *p* ≤ 0.05 and ** *p* ≤ 0.01. (**b**) Representative images of metastatic nodules are shown for each mouse’s lungs. (**c**) Representative images of the resected tumors.

Our previous study demonstrated that the TGFβ stimulation of melanoma cells suppressed cell migration in vitro [12]. Furthermore, as shown in Figure 4d, blocking the Smad pathway in our orthotopic transplantation model led to an increased liver metastatic burden. Thus, these results suggest that blocking TGFβ/Smad signaling in vivo could also regulate the metastatic dissemination of melanoma cells to distant organs. To address this, we used a preclinical model of melanoma lung colonization [44,45,46]. Briefly, as described in Figure 5a, Smad3 CRISPR-KO, Smad4 CRISPR-KO and control NT CRISPR-KO a375m melanoma cells were injected intravenously into NSG mice (tail vein injection; 8 mice/group).

Twenty-one days post injection, animals were sacrificed and lungs were resected before being stained in Bouin solution, as previously described [45]. Interestingly, as shown in Figure 5b, both Smad3 and Smad4 CRISPR-KOs showed a strong increase in numbers of metastatic lung lesions compared to control animals. Figure 5c shows representative images of the resected tumors. These results indicates that inhibition of the TGFβ/Smad canonical signaling pathway not only increased primary tumor growth but also significantly increased the metastasis burden. They are also consistent with our results from the spontaneous liver metastasis preclinical model (Figure 4d). Altogether, our data define the TGFβ/Smad signaling axis as a potent suppressor of metastasis.

## 4. Discussion

In this study, we investigated the role of TGFβ in stem cell maintenance in melanoma and the relationship with the TGFβ/Smads signaling axis in tumorigenesis and metastasis. We found that TGFβ inhibits stem cell maintenance in several human cutaneous cell lines. Furthermore, we found that TGFβ acts as a potent tumor suppressor, blocking primary tumor formation but also as a strong suppressor of metastasis, preventing the spread and development of secondary liver and lung metastatic nodules in vivo. Our data are in agreement with and support our previous in vitro work showing that TGFβ acts as an anti-migratory factor in melanoma [12,13]. They underscore TGFβ and Smad signaling as potent regulators implicated in self-renewal as well as suppressors of both tumor formation and metastasis in cutaneous melanoma.

Melanoma stem cells have many capabilities compared to differentiated cells, such as self-renewal, differentiation, plasticity, immune evasion, drug resistance and the promotion of cell migration and metastasis. A study showed that melanoma CSCs secreted factors can activate neutrophils and support cancer progression, therefore increasing the importance of the interplay between tumor microenvironment and cancer progression [47]. Indeed, soluble factors such as TGFβ can modify the tumor microenvironment. Such mechanisms implicating CSCs are directly associated with melanoma progression, metastasis and tumor heterogeneity [48]. Thus, our data defining TGFβ as an inhibitor of CSC self-renewal is consistent with a role of TGFβ as an inhibitor of tumor formation, progression, and metastasis. Moreover, in future studies, it will be interesting to further characterize the precise role of TGFβ signaling on stemness, using in vivo and in vitro diluting limiting assay.

In melanoma, several stem cell markers are expressed in subpopulations of CSCs which exhibit increased tumor potential. One of the first identified CSC marker is CD133, which is an extracellular protein linked to a subset of melanoma cells displaying stem-cell like properties and increased tumorigenicity [22]. Isolated subpopulations of melanoma cells expressing CD133 are more proliferative and more invasive than their CD133-negative counterparts [49,50]. Furthermore, CD133 was also found to be expressed in metastatic extract from melanoma patients, which is consistent with a role for CSC in promoting metastasis [22]. Another CSCs subpopulation is characterized by the ALDH+ melanoma cells. In particular, the ALDH1A1 and ALDH1A3 isoenzymes were shown to be enriched in melanoma tumors [41]. In this study, we found that TGFβ inhibits CSCs’ self-renewal capacity in multiple melanoma cell lines. We also show that TGFβ efficiently reduces the percentage of several of the main CSC subpopulations, CD133+, ALDHA1 and ALDHA3. These potent effects inhibiting self-renewal ability likely reflect the strong tumor-suppressor role played by TGFβ in these tumors. These results are also in line with what was observed in other types of solid tumors, such as pancreatic cancer, where Smad4 upregulation was found to be inversely correlated with ALDHA1 expression [51]. They suggest that TGFβ/Smad signaling may exert anti-CSC self-renewal activity on a broader range of tumors than melanoma alone.

Interestingly, while the TGFβ effects on melanoma cancer stem cell maintenance require the Smad pathway, they also appear to be Smad3/4 specific and Smad2-independent. Such Smad2 or Smad3 specificity downstream of TGFβ signaling has been reported in the context of other cancer-related mechanisms [52,53,54,55,56]. For instance, the E1A-like inhibitor of differentiation-2 (EID-2) protein can suppress TGFβ signaling by specifically blocking the TGFβ-induced formation of Smad3–Smad4 complexes [54]. Another study showed that Smad3 silencing in keratinocytes interfered with growth inhibition while Smad2 silencing had no phenotypic effect [56]. Our group also previously showed that menin, a potent tumor suppressor, specifically interacts with Smad3 to mediate TGFβ anti-proliferative responses in pituitary adenoma [52]. Furthermore, previous work from our laboratory and others also showed that TGFβ-mediated inhibition of telomerase activity and cell immortalization relies on Smad3 signaling independently of Smad2 [53,55]. A previous study showed that the constitutive phosphorylation of the Smad3 linker region by MAPK and CDK/GS3 modulates TGFβ-mediated resistance to cell cycle arrest by interfering with p15 and p21 [57]. Thus, phosphorylation on distinct specific sites of the Smads can lead to differential regulation of the cell cycle. Altogether, these studies are consistent with our present findings in melanoma, suggesting that Smad3 may play a more prominent role in the mediation of the TGFβ tumor-suppressive effects compared to Smad2 in various models of solid tumors.

Phenotype switching refers to the switch from a proliferative to an invasive phenotype, conferring plasticity to cancer cells. The switch implicates transcriptional reprogramming involving a panoply of signaling pathways with their respective downstream regulators including TGFβ/SMADs, Hippo/TAP/TAZ and Wnt/B-catenin [58]. Furthermore, MITF (microphthalmia-associated transcription factor) is an important melanocytic lineage-specific transcription factor also associated with phenotype switching. Indeed, MITF low expression is correlated with invasiveness and high expression is correlated with a more proliferative phenotype [59]. TGFβ has been shown to inhibit the MITF transcription through repressed protein kinase A activity, which is therefore correlated with the invasiveness phenotype of TGFβ [60]. In parallel, TGFβ has been shown to exert a dual role during cancer progression in some types of cancer [16,61]. While inducing tumor suppression in normal epithelial cells and early carcinomas, TGFβ promotes metastasis in more advanced stages of cancer [16,62,63,64].

However, the TGFβ function in melanoma remains controversial. While previous studies showed that overexpression of the TGFβ signaling inhibitor SMAD7 reduced the proliferation and metastatic potential of the 1205Lu melanoma cell line [65,66], other studies suggested that TGFβ itself could inhibit tumor cell migration and metastasis [12,13]. Interestingly, the 1205Lu melanoma cell line used in the former studies [60,65,66] was not responding to TGFβ in the tumorsphere assays performed in our study, which could explain the differential TGFβ outcome observed in other melanoma cell lines. A separate study showed that a recombinant cytotoxin (cytotoxin-II) indirectly inhibited SMAD2/3 mRNA expression and correlated with increased caspase 8 and 9 in vitro [67]. However, these results, using an indirect inhibitory approach, were not confirmed in vivo. In contrast, our results clearly indicate that direct TGFβ silencing using SMAD KOs significantly reduced proliferation, tumorigenesis and metastasis both in vitro and in vivo.

We previously found TGFβ to inhibit cell migration and invasion in vitro in several models of melanoma [12]. The present study is in accordance with these results and clearly indicates that TGFβ/Smad signaling prevents tumor progression in vivo, using preclinical models of melanoma metastasis. They are also consistent with a role for TGFβ as an inhibitor of CSC self-renewal, further highlighting TGFβ as an anti-metastatic factor in melanoma.

## 5. Conclusions

Finally, having shown that TGFβ inhibits stemness and prevents tumor formation, progression and metastasis, our study underscores the potential for using TGFβ-mimicking or stimulating agents as new therapeutics for cutaneous melanoma. For instance, avotermin, a recombinant TGFβ3 used in clinical trials for the prophylactic treatment of tissue scarring of the skin, could be tested for the treatment of melanoma patients [68,69]. In addition, the Vitamin E derivative δ-tocotrienol was shown to exert a specific anti-tumor activity against melanoma CSCs [70] and as such could be tested in combination treatment with TGFβ to target specifically melanoma stem cells. Our findings, which highlighted the complex role of the TGF-β pathway in melanoma tumorigenesis and metastasis, could pave the way for novel therapeutic approaches targeting this growth factor for cancer inhibition.

## Figures and Tables

**Table 1:** Primer sequences for CRISPR Knock-out cloning.

Primer Name	Single-Guide Primer Sequence
scrsg1-F	5′-CACCGACGGAGGCTAAGCGTCGCAA-3′
scrsg1-R	5′-AAACTTGCGACGCTTAGCCTCCGTC-3′
scrsg2-F	5′-CACCGCGCTTCCGCGGCCCGTTCAA-3′
scrsg2-R	5′-AAACTTGAACGGGCCGCGGAAGCGC-3′
scrsg3-F	5′-CACCGATCGTTTCCGCTTAACGGCG-3′
scrsg3-R	5′-AAACCGCCGTTAAGCGGAAACGATC-3′
Smad2sg4-F	5′-CACCGTGGCGGCGTGAATGGCAAGA-3′
Smad2sg4-R	5′-AAACTCTTGCCATTCACGCCGCCAC-3′
Smad3sg2-F	5′-CACCGTTCACGATCGGGGGAGTGAA-3′
Smad3sg2-R	5′-AAACTTCACTCCCCCGATCGTGAAC-3′
Smad4sg1-F	5′-CACCGAACTCTGTACAAAGACCGCG-3′
Smad4sg1-R	5′-AAACCGCGGTCTTTGTACAGAGTTC-3′

**Table 2:** Primer sequences for qPCR.

Primer Name	Primer Sequence for qPCR
CD133-F	TACCAAGGACAAGGGGTTCAC
CD133-R	CAGTCGTGGTTTGGCGTTGTA
ABCG2-F	GCTCAGGAGGCCTTGGGATA
ABCG2-R	GGCTCTATGATCTCTGTGGCTTT
ALDH1A1-F	CTGTGTTCCAGGAGCCGAAT
ALDH1A1-R	CTGCCTTGTCAACATCCTCCTTA
ALDH1A3-F	GGAAGAAGGAGATAAGCCCGAC
ALDH1A3-R	AGCCCTCCAGGTCGATGAAA
GAPDH-F	GACAGTCAGCCGCATCTTCT
GAPDH-R	GCGCCCAATACGACCAAATC

## Data Availability

The data that support the findings of this study are available from the corresponding author J.B. upon reasonable request.

## Supplementary Materials

The following supporting information can be downloaded at: https://www.mdpi.com/article/10.3390/cancers16010224/s1, Figure S1: Original Western-blotting images; Table S1: The sequence of genes in the manuscript.
