# Supplementary material for: Transforming Growth Factor-β/Smad Signaling Inhibits Melanoma Cancer Stem Cell Self-Renewal, Tumor Formation and Metastasis
Source: Cancers (Basel). 2024 Jan 3;16(1):224. doi: 10.3390/cancers16010224 (PMC10778361; doi:10.3390/cancers16010224)

**Supplementary Table S1.** The sequence of genes in the manuscript.

| | |
|---|---|
| # Sequence | TGGCGGCGTGAATGGCAAGATGG |
| # Genome | hg19 |
| # PAM | NGG |
| # Position | chr18:45423097-45423120:+ |
| # Version | CRISPOR 5.01, 2023-08-31T19:44:01CEST |
| Chromosomal position of sgRNA binding site | Chr18:47896727-47896749 |
| #guideId | targetSeq |
| **SMAD2 sg4** | TGGCGGCGTGAATGGCAAGATGG |
| | |
| # Sequence | TTCACGATCGGGGGAGTGAAAGG |
| # Genome | hg19 |
| # PAM | NGG |
| # Position | chr15:67358507-67358530:- |
| # Version | CRISPOR 5.01, 2023-08-31T19:48:51CEST |
| Chromosomal position of sgRNA binding site | Chr15:67066170-67066192 |
| #guideId | targetSeq |
| **SMAD3 sg2** | TTCACGATCGGGGGAGTGAAAGG |
| | |
| # Sequence | AACTCTGTACAAAGACCGCGTGG |
| # Genome | hg19 |
| # PAM | NGG |
| # Position | chr18:48593460-48593483:- |
| # Version | CRISPOR 5.01, 2023-08-31T19:50:26CEST |
| Chromosomal position of sgRNA binding site | Chr18:51067091-51067113 |
| #guideId | targetSeq |
| **SMAD4 sg1** | AACTCTGTACAAAGACCGCGTGG |
| | |

**Supplementary Table S1.** *Cont.*

| mitSpecScore | cfdSpecScore | offtargetCount | targetGenomeGeneLocus | Doench '16-Score | OldDoench '16-Score |
|---|---|---|---|---|---|
| 81 | 89 | 84 | exon:SMAD2 | 60 | 59 |

[redacted]

| mitSpecScore | cfdSpecScore | offtargetCount | targetGenomeGeneLocus | Doench '16-Score | OldDoench '16-Score |
|---|---|---|---|---|---|
| 87 | 92 | 42 | exon:SMAD3 | 53 | 52 |

[redacted]

| mitSpecScore | cfdSpecScore | offtargetCount | targetGenomeGeneLocus | Doench '16-Score | OldDoench '16-Score |
|---|---|---|---|---|---|
| 94 | 95 | 39 | exon:SMAD4 | 70 | 75 |

[redacted]

**Supplementary Table S1.** *Cont.*

| Chari-Score | Xu-Score | Wu-Crispr-Score | Doench '14-Score | Wang-Score | Moreno-Mateos-Score |
|---|---|---|---|---|---|
| 84 | 0.16579 | 67 | 24 | 66 | 66 |

[redacted]

| Chari-Score | Xu-Score | Wu-Crispr-Score | Doench '14-Score | Wang-Score | Moreno-Mateos-Score |
|---|---|---|---|---|---|
| 63 | -0.1942 | 0 | 19 | 24 | 63 |

[redacted]

| Chari-Score | Xu-Score | Wu-Crispr-Score | Doench '14-Score | Wang-Score | Moreno-Mateos-Score |
|---|---|---|---|---|---|
| 97 | 0.30044 | 87 | 92 | 81 | 24 |

[redacted]

**Supplementary Table S1.** *Cont.*

| Azimuth in-vitro-Score | CCTop-Score | Out-of-Frame-Score | Lindel-Score | GrafEtAlStatus | grafType |
|---|---|---|---|---|---|
| 55 | 67.249906 | 72 | 84 | GrafOK | |

[redacted]

| Azimuth in-vitro-Score | CCTop-Score | Out-of-Frame-Score | Lindel-Score | GrafEtAlStatus | grafType |
|---|---|---|---|---|---|
| 59 | 48.7785995 | 60 | 82 | GrafOK | |

[redacted]

| Azimuth in-vitro-Score | CCTop-Score | Out-of-Frame-Score | Lindel-Score | GrafEtAlStatus | grafType |
|---|---|---|---|---|---|
| 20 | 47.7187295 | 64 | 90 | GrafOK | |

[redacted]

# **Figure S1.** Original Blots: Cropped regions are in red

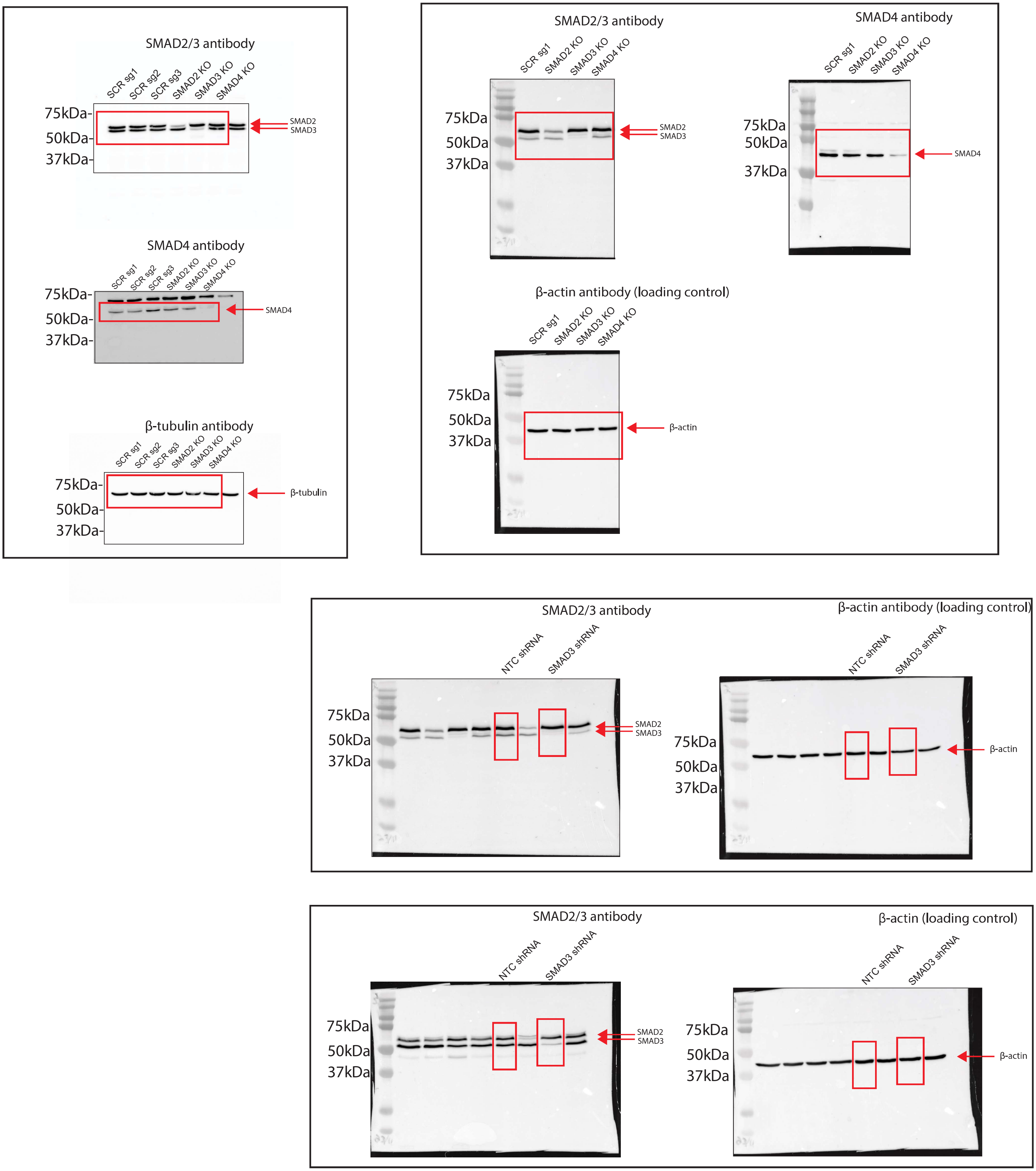

Supplement: Supplementary file 1 [file cancers-16-00224-s001.zip › cancers-2785143-supplementary.pdf]
